# Dilated Cardiomyopathy, De Novo Heart Failure, and Cardiogenic Shock With End-Organ Failure in a Patient With No Cardiac History Following a Trial of Amitriptyline

**DOI:** 10.7759/cureus.67374

**Published:** 2024-08-21

**Authors:** Zachary S Kauffman, Melaku Demede

**Affiliations:** 1 Research, DeBusk College of Osteopathic Medicine, Lincoln Memorial University, Beckley, USA; 2 Cardiology, Beckley Appalachian Regional Hospital, Beckley, USA

**Keywords:** tricyclic antidepressants, cardiogenic shock, heart failure, amitriptyline, dilated cardiomyopathy

## Abstract

A 69-year-old female with no cardiac history presented with dilated cardiomyopathy and de novo congestive heart failure, with an ejection fraction of less than 20%. This patient had struggled over the prior six weeks with exacerbation of chronic obstructive pulmonary disease complicated by pneumonia and as such had taken several trials of antibiotics. Four days prior to her presentation, she was prescribed amitriptyline by her primary care physician to help with sleep. Two days after the presentation, she developed cardiogenic shock secondary to acutely decompensated heart failure. End-organ dysfunction presented as aspartate aminotransferase (AST) and alanine aminotransferase (ALT) of greater than 1000 U/L and a glomerular filtration rate (GFR) as low as 23 mL/min. Cardiac catheterization showed non-obstructive coronary artery disease, and cardiac MRI showed an ejection fraction of 14%. She was discharged 14 days after her initial presentation with a diagnosis of NYHA Class 3 Stage C acute systolic heart failure with dilated cardiomyopathy.

## Introduction

Tricyclic antidepressants are a class of drugs commonly used to treat depression. Tricyclic antidepressants such as amitriptyline have well-known cardiotoxic effects, including malignant arrhythmias and sudden cardiac death [1]. More rarely, these drugs may cause myocarditis and dilated cardiomyopathy; this interaction is decidedly less often documented in the literature. One such case resulted from the long-term use of amitriptyline [2], and another resulted from an intentional overdose of amitriptyline [2,3]. The mechanism of injury of drugs in this class in causing cardiomyopathy has not yet been elucidated.

## Case presentation

A 69-year-old female with a history of COPD and no cardiac history presented to the emergency department (ED) with complaints of shortness of breath. In the ED, vital signs were significant for a pulse of 111 beats per minute, a respiratory rate of 20 breaths per minute, and a blood pressure of 123/85 mmHg. The patient’s mentation was waxing and waning upon presentation, but she detailed that over the last six weeks, she had some cough and chest congestion. Her primary care provider (PCP) had put her on antibiotics for pneumonia, which helped initially. Two weeks prior to this presentation, she was feeling bad again and visited her PCP again, who again initiated antibiotics in addition to starting steroids. She reports that a CT chest taken at that time was normal. Four days prior, she visited her PCP again for continued shortness of breath and difficulty sleeping. At that time, her PCP began montelukast 10 mg and amitriptyline 100 mg. This caused nausea, vomiting, diarrhea, and anorexia for the following four days leading up to her presentation to the ED. In the ED, she denied hematochezia or hemoptysis. Physical examination was significant only for pallor. The skin was warm and dry, and there was no wheezing, rales, or rhonchi. There was no jugular venous distention or murmurs noted. There was no edema noted. She was admitted to the hospital, and cardiology was consulted.

**Figure 1:**
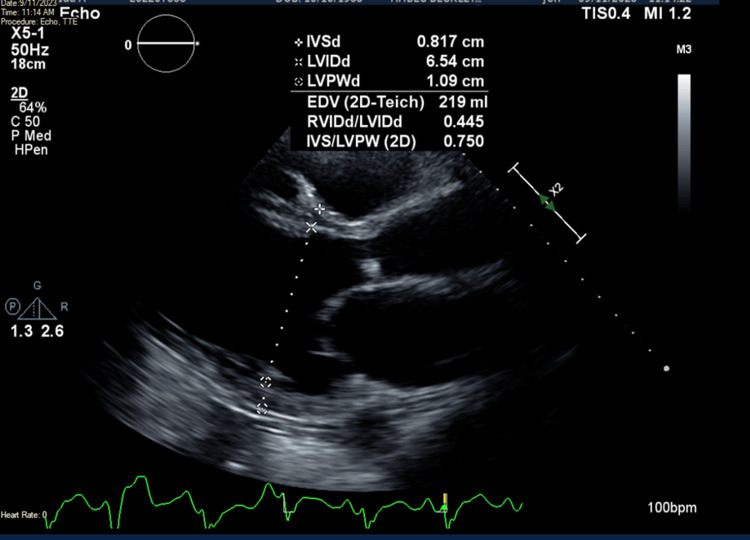
Transthoracic echocardiogram showing left ventricular diastolic dimension of 6.54 cm.

**Figure 2:**
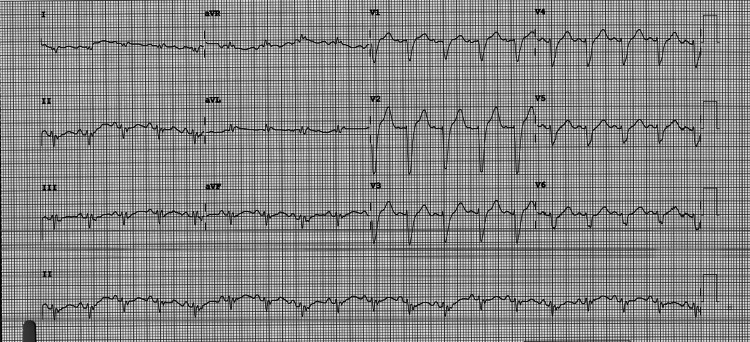
12-lead EKG showing sinus tachycardia with some ST-T abnormalities, early repolarization versus ischemia in anterior leads and lateral leads. EKG, electrocardiography

Upon admission, her temperature was 97.5°F, pulse was 110 beats per minute, respiratory rate was 18 breaths per minute, and blood pressure was 135/85 mmHg. Troponin I was 316 ng/L and NT-pro-BNP was 23,985 pg/L. Lactic acid was also elevated at 3.6 mmol/L and procalcitonin was elevated at 0.27 ng/L. Aspartate aminotransferase (AST) and alanine aminotransferase (ALT) were 1,087 U/L and 1,069 U/L, respectively. Blood urea nitrogen (BUN) was 73 mg/dL, and creatinine was 1.89 mg/dL with a BUN/creatinine ratio of 38.6. Glomerular filtration rate (GFR) was 26.4 ml/min. Transthoracic echocardiography (TTE) showed an ejection fraction of 20-25%, global hypokinesis, and dilated cardiomyopathy (Figure 1). Specifically, the left ventricular diastolic dimension was 6.54 cm, while the right ventricular diastolic dimension was 2.91 cm. TTE also showed a potential left ventricular thrombus, so therapeutic heparin was begun. 12-lead EKG showed sinus tachycardia with a rate of 109 beats per minute with some ST-T abnormalities, early repolarization versus ischemia in anterior leads and lateral leads (Figure 2). QRS complexes were of normal duration, and QTc was prolonged. Given the EKG findings and the patient’s vitals, diagnoses of non-ST-elevated myocardial infarction and sepsis were made; however, urinalysis and chest X-ray failed to reveal a source of infection at that time.

The following day, NT-pro-BNP and troponin I were trending down to 20,618 pg/L and 98 ng/L, respectively. AST and ALT were trending up to 1,763 U/L and 1,885 U/L, respectively. Her BUN was 85 mg/dL and her creatinine was 2.12 mg/dL, with a BUN/creatinine ratio of 40.1 and a GFR of 23.1 mL/min. Her lactic acid was 3.6 mmol/L in the morning and increased to 4.0 mmol/L by evening. Physical examination was benign, and the patient was alert and oriented. The following morning, NT-pro-BNP and troponin I continued to trend down to 13,671 pg/L and 84 ng/L, respectively. Her AST and ALT continued to trend up to 1,802 U/L and 2,265 U/L. Her lactic acid remained at 4.0 mmol/L. That morning, her mentation was grossly altered, as the patient was oriented to person but not to time or place. Her blood pressure dropped to 87/68 mmHg by mid-morning, and her extremities were cold to the touch. There was no jugular venous distention noted. Dorsalis pedis pulses were 1+ bilaterally. Levophed was initiated and the diagnosis of septic shock was made, meanwhile, no source of infection had been identified. Transfer to an external facility was initiated for a shock with end-organ damage and possible left ventricular thrombus found on TTE the day before.

**Figure 3:**
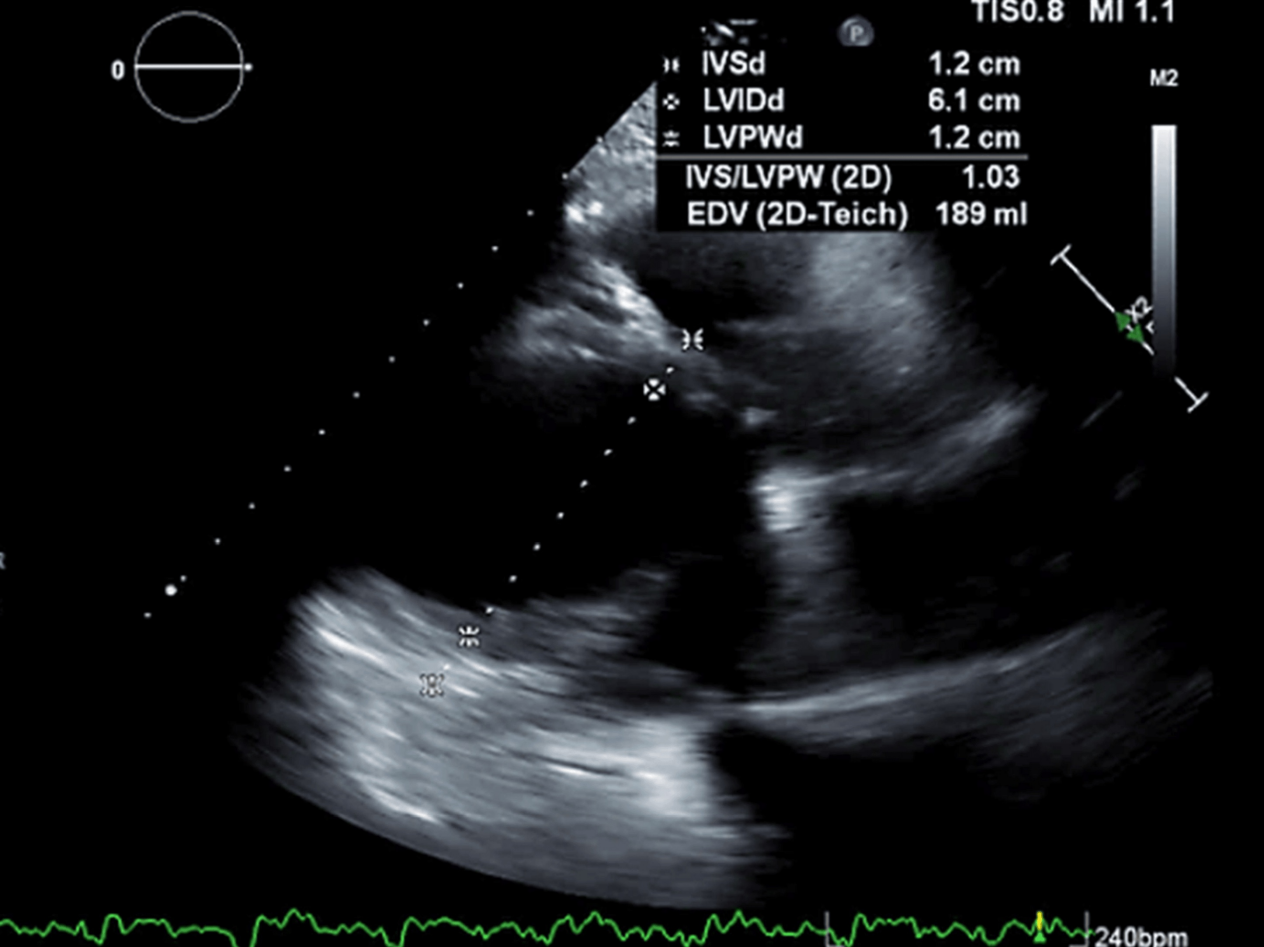
Transthoracic echocardiogram showing left ventricular diastolic dimension of 6.1 cm.

At the external facility, the patient was admitted to the cardiac intensive care unit (ICU). The patient was lethargic and with jugular venous distention on the exam. She was diuresed and begun on oral anticoagulation for possible left ventricular thrombus. Repeat TTE showed an echogenic lesion in the mid-lateral left ventricle as well as hypertrophied papillary muscles (Figure 3). She was started on dobutamine, vasopressin, milrinone, and levophed. Milrinone was discontinued the next day.

**Figure 4:**
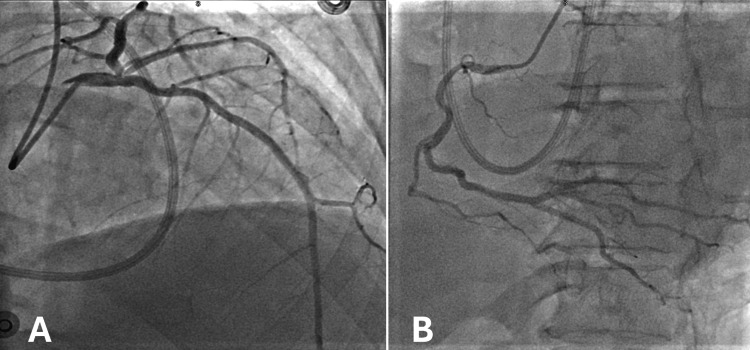
Coronary angiography showing non-occlusive coronary artery disease in the left (A) and right (B) coronary artery systems.

**Figure 5:**
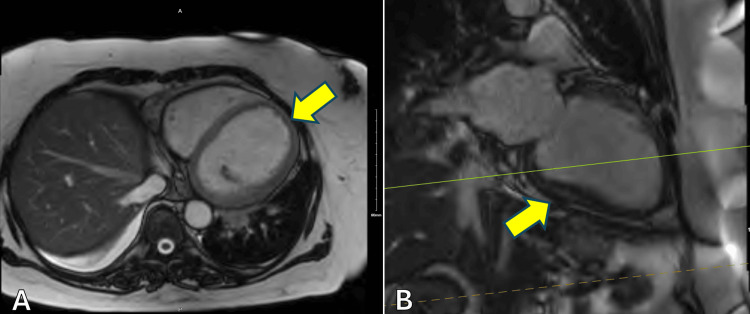
Cardiac MRI showing a dilated left ventricle with no left ventricular thrombus in axial (A) and sagittal (B) views. Left ventricle indicated by the yellow arrow. MRI, magnetic resonance imaging

One day later, upon assessment by cardiology, her cardiac index was 1.6 L/min/m^2^, her cardiac output was 3.5 L/min, and her central venous pressure was 5 mmHg, with pulmonary arterial pressure of 36/22 mmHg. She was then restarted on milrinone for cardiogenic shock secondary to biventricular failure. Lactic acid that day was normal at 1.7 mmol/L, trending down from 4.0 mmol/L three days prior. Over the next several days, the patient’s condition stabilized. The patient underwent cardiac catheterization on day eight following her initial ED visit, which revealed only nonobstructive coronary artery disease (Figure 4). No percutaneous coronary intervention was performed. Subsequent cardiac magnetic resonance imaging (MRI) three days later ruled out a left ventricular thrombus (Figure 5). The ejection fraction on cardiac MRI was 14%. Anticoagulation was discontinued. Per the radiographic report, the cardiac MRI was limited due to the patient's request for early termination of the study.

The patient was discharged 12 days after initial presentation to the ED with the diagnoses of cardiogenic shock, resolved; acute systolic heart failure with dilated cardiomyopathy, NYHA Class 3 Stage C; pulmonary hypertension; nonobstructive coronary artery disease; and fatty liver disease. The patient refused LifeVest placement and was begun on guideline-directed medical therapy (GDMT). She was discharged on Farxiga and metoprolol, and she was recommended to follow up with cardiology.

The patient was seen at an outpatient cardiology clinic six weeks after her initial presentation to the ED. She reported that she was feeling much better and denied shortness of breath, orthopnea, or lower extremity swelling. She also reported that she was not taking her Farxiga or her metoprolol. Physical examination was benign. In another outpatient follow-up at the same clinic six weeks later, she stated that she was feeling well and reported that she was still not taking her metoprolol.

**Figure 6:**
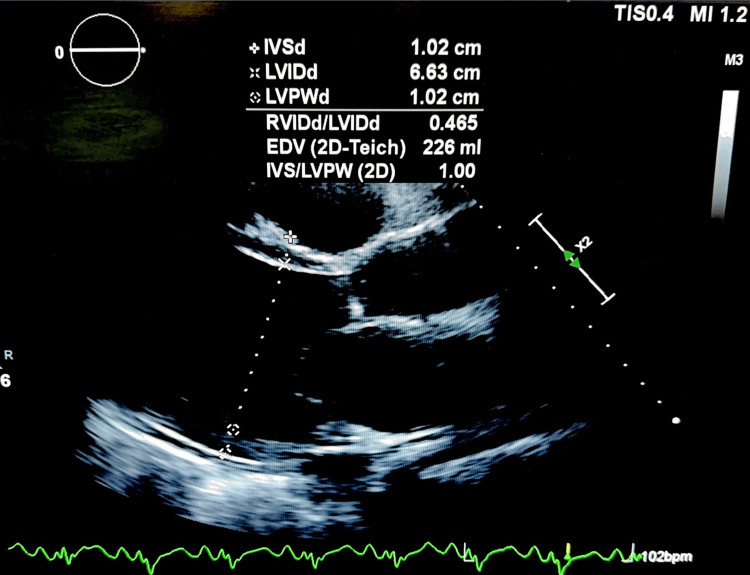
Transthoracic echocardiogram showing left ventricular diastolic dimension of 6.63 cm.

The patient then presented back to the original ED less than a month later. She reported worsening shortness of breath. An echocardiogram at that time showed a left ventricular diastolic dimension of 6.63 cm and a right ventricular diastolic dimension of 3.08 cm (Figure 6). Left ventricular ejection fraction was 20-25%.

## Discussion

This was a patient with no cardiac history who presented with de novo systolic CHF and dilated cardiomyopathy that progressed to cardiogenic shock with end-organ failure. Notably, the patient had been started on montelukast and amitriptyline only four days prior to arrival. A review of the literature on amitriptyline-induced dilated cardiomyopathy reveals two case studies. In the first, an elderly woman had been taking amitriptyline for years before presenting with signs of myocarditis and dilated cardiomyopathy, which was confirmed on autopsy [2]. In the second, the presenting patient had attempted suicide by amitriptyline overdose [3].

While the interaction between tricyclic antidepressants and dilated cardiomyopathy is rare, evidence suggests it is not unheard of. A case/noncase analysis of adverse drug reactions (ADRs) documented in the French PharmacoVigilance Database from January 1st, 1990 to June 30th, 2007 showed that the tricyclic antidepressants clomipramine and amitriptyline were associated with 1,418 and 1,129 ADRs, respectively [4]. Of these ADRs, clomipramine and amitriptyline were respectively associated with only four and three cases of dilated cardiomyopathy. These findings suggest that, while rare, dilated cardiomyopathy as a result of tricyclic antidepressant use may represent an idiosyncratic ADR, with a small number of the population being predisposed to this reaction.

Furthermore, at least one study suggests that, upon removal of tricyclic antidepressants, the resultant dilated cardiomyopathy did resolve [5]. In the two patients detailed in this study, both saw echocardiographic evidence of normalization of left ventricular diameters upon discontinuation of tricyclic antidepressant treatment. While we cannot say that tricyclic antidepressants play a causal role in the development of dilated cardiomyopathy, a similar trajectory was seen in our patient - repeat echocardiogram in December of 2023, three months after initial presentation, revealed an ejection fraction of 20-25%. This is increased from the lowest ejection fraction measured by echocardiography at 15% during the initial episode.

It is too early to ascertain whether ventricular diameter will return to normal, a problem further complicated by the lack of baseline echocardiography on this patient. Furthermore, any structural normalization is likely slowed by the fact that she has reported noncompliance with her Farxiga and metoprolol, both of which are associated with preventing and even reversing cardiac remodeling [6,7]. However, the current trajectory does suggest that normalization of cardiac size and function may still be possible.

## Conclusions

On the whole, the current case study details acute-onset heart failure with dilated cardiomyopathy resulting in cardiogenic shock in a patient with no cardiac history after trialing a tricyclic antidepressant. Case studies are inherently limited in their ability to infer causation, and as such we cannot say that the tricyclic antidepressant was the cause of her presentation. This is compounded by the fact that the patient has no cardiac history and had no echocardiogram prior to starting the trial of amitriptyline. Thus, the patient may have had an underlying heart condition that was exacerbated by a number of factors. Furthermore, given the patient’s noncompliance and lack of medical history, it is difficult to say what predisposed her to such a reaction. That being said, the current case study does add to the relatively small body of literature on dilated cardiomyopathy as a potential side effect of tricyclic antidepressants. Future research should investigate this link, paying particular attention to genetic and historical factors that may predispose patients to this interaction.
